# Standardised assessment of personality – a study of validity and reliability in substance abusers

**DOI:** 10.1186/1471-244X-8-7

**Published:** 2008-01-25

**Authors:** Morten Hesse, Joachim Rasmussen, Mads Kjær Pedersen

**Affiliations:** 1 Aarhus University, Centre for Alcohol and Drug Research, Copenhagen Division, Købmagergade 26E, 1150 Copenhagen C, Denmark; 2 City of Copenhagen, Inner City Rehabilitation and Counselling Centre, Treatment Unit, Hørsholmsgade 20A, 2200 Copenhagen N, Denmark

## Abstract

**Background:**

Brief screening instruments for co-morbid personality disorders could potentially have great value in substance abuse treatment settings.

**Methods:**

We assessed the psychometric properties of the 8-item Standardised Assessment of Personality – Abbreviated Scale (SAPAS) in a sample of 58 methadone maintenance patients.

**Results:**

Internal consistency was modest, but similar to the original value (alpha = 0.62), and test-retest correlation at four months follow-up was moderately encouraging for a short instrument such as this (n = 31, test retest intraclass correlation = 0.58), and change at the mean level was minimal, but marginally significant (from an average of 3.3 to 3.8, p = 0.06). Analyses of nurse ratings of patients' behaviour at the clinic showed that SAPAS was significantly correlated with nurse ratings of externalizing behaviour (r = 0.42, p = 0.001), and Global Assessment of Functioning (r = -0.36, p = 0.006), but unrelated to intoxication (r = 0.02, NS), or withdrawal (r = 0.20, NS).

**Conclusion:**

There is evidence that the SAPAS is a modestly valid and relatively reliable brief screening measure of personality disorders in patients with ongoing substance abuse undergoing methadone maintenance. It can be used in situations where limited resources are available, and researchers or others wish to get an impression of the degree of personality pathology in a clinical population, as well as for screening purposes.

## Background

Personality disorders are among the most common co-morbidities among patients with substance use disorders [1,2]. Personality disorders complicate treatment in a range of ways, elicit negative emotional reactions in clinicians [3], and are associated with worse outcome in treatment for substance use disorders [4-7]. Evidence is emerging that substance abuse treatment is more effective, if it addresses personality related issues, including personality disorders [8-10].

However, identifying patients with co-morbid personality disorders remain a challenge for substance abuse treatment services. Self-report inventories tend to diagnose nearly all substance abusers with personality disorders [11], and often traits improve rapidly with time [12,13]. Diagnostic interviews are time-consuming, and require substantial training, and are therefore expensive for service providers to carry out. Reducing the number of patients who need a full personality disorder examination by screening out patients without personality disorders could therefore be attractive. However, inexpensive, efficient and valid measures of personality disorder have not been validated for substance abusing populations.

Only three interviewer-administered screens for personality disorder have been published. Langbehn and colleagues [14] have developed the Iowa Personality Disorder Screen (IPDS) to provide a mini-structured interview that the authors estimate can be completed in five minutes. The IPDS consists of eleven questions that address general personality disorder criteria as well as specific criteria. The instrument has been validated against the Structured Interview for DSM-IV Personality Disorders (SIDP-IV). The authors reported excellent sensitivity (92%) and good specificity (79%), although the validation was a somewhat circular exercise, as the IPDS items were derived from the SIDP-R. A structured patient interview for personality disorders, the Rapid Personality Assessment Schedule, requires staff training and performs moderately well as a screen for personality disorder when compared to the full version of the PAS (sensitivity = 64%, specificity = 82%) [15]. Moran and colleagues developed the Standardised Assessment of Personality, Abbreviated Scale (SAPAS), a brief interviewer-administered screener that could be completed in less than 2 minutes, and reported good sensitivity (0.94) and specificity (0.85) in a sample of psychiatric patients with a range of different disorders [16].

Such a brief measure could potentially function as a screener, that is, a brief instrument that could be administered in minutes as part of an intake assessment, and could alert clinicians to the possibility of personality disorder, and refer to further assessment. Also, when doing audits of the prevalence of personality disorders in various clinical populations, or screening for research studies on personality disorders, a very brief screener might save costs.

In this study, we assessed the validity and reliability of the SAPAS in patients undergoing opioid substitution treatment in a low-threshold setting. We used nurse ratings of patients' behaviour to indicate the concurrent validity of the SAPAS scores. Given that problems with behavioural inhibition, emotional problems, and problems controlling substance use characterize personality disorders, we chose to use observations of the patients' behaviour at medication pick-up as validation of the instruments.

## Methods

### Setting

The setting was a public opioid substitution clinic in the City of Copenhagen. The clinic is one of four clinics providing free substitution treatment for all opioid dependent patients, who are referred for opioid substitution treatment within the clinics uptake area. The staff at the clinic are social workers, physicians, nurses, healthcare assistant, psychologist, and administrative staff. No requirement is made that patients abstain from other drug or alcohol use during treatment, but attempts are made to help patients stabilize drug and alcohol problems during treatment.

Patients who are violent or threatening to an extent that is not manageable within the clinic are referred to a special opioid substitution bus run by a private company. Thus, patients are practically never discharged from treatment or detoxified against their wish. Pharmacotherapy works with flexible and individualized dosing in close collaboration between physician and patient. There is no official maximum dose of methadone or buprenorphine, although generally physicians try to keep doses under 150 millilitres per day. All treatment is free, and is available for patients with legal residence in Denmark who either live in the City of Copenhagen, or are homeless and mainly stay in the Copenhagen area. Tapering of medication is only carried out on patient demand, and is generally neither encouraged nor discouraged. Caseworkers are assigned to clients, and provide counselling and typical case management services (e.g., assessment of problems and needs, planning and setting goals, linking with other services, advocating the patients case with external partners), but are not responsible for monitoring patients' progress, as patients are expected to come for help when they need it.

Patients are offered access to drop-in centres that are placed in various areas of Copenhagen, but no drop-in centre or café is available at the clinics.

Nurses at the Inner City centre do health check-ups with patients, during which they assess needs for medical treatment, plan for treatment, contraception, hepatitis testing, HIV-testing, hepatitis immunization, and the clinic physician can refer patients to testing or treatment at community hospitals.

### Procedures

Patients were approached in the clinic by the first author, and asked whether they were willing to consider participating in a research study concerning their treatment. Following this, they were given written and verbal information about the purposes of the study. The main purpose of the study was a quasi-experimental study of teaching knowledge about personality disorders to case-workers. A secondary purpose was to test psychometric properties of instruments. At follow-up, patients were followed up by in-person (n = 29) or telephone interviews (n = 2). Different interviewers at both points interviewed a total of 23 subjects, and the same interviewer interviewed 8 patients.

At the follow-up interview, approximately 4 months after the first assessment, patients were again administered the SAPAS (see below). When the SAPAS was administered the second time, patients were instructed not to try to remember what they had said the first time, and were told that we wanted to know "if people see themselves in the same way at two different points in time".

### Measures

At baseline, patients were administered a very brief 5 minute interview.

#### The Standardised Assessment of Personality – Abbreviated Scale (SAPAS)

The SAPAS is a brief interview-based screening instrument consisting of eight dichotomously rated items taken from the opening section of an informant-based interview, the Standardised Assessment of Personality, and has been found to have high sensitivity and specificity as a screener for personality disorders [16]. The items are listed in Table 1.

**Table 1:** Reliability of the SAPAS

	Four months test-retest reliability		Internal consistency	
	Kappa	Kappa with different interviewers	Item-total correlation	Alpha if deleted

N	31	23	58	
Difficulty making and keeping friends	0.53	0.47	0.41	0.57
Usually a loner	0.58	0.51	0.31	0.60
Trusts people	0.58	0.53	0.47	0.55
Normally loses temper easily	0.32	0.32	0.27	0.61
Normally impulsive	0.50	0.58	0.20	0.63
Normally a worrier	0.26	0.25	0.30	0.60
Normally dependent	0.32	0.27	0.35	0.59
Perfectionist	0.50	0.57	0.29	0.61

#### Nurse ratings of patient functioning

Nurses rated patients on medication pick-ups using four rating scales developed for the study. The four rating scales represented intoxication (0–4, with 0 representing no intoxication and 4 severe intoxication), withdrawal (0–4, with 0 representing no withdrawal and 4 severe withdrawal), externalizing behaviour (0–4, with 0 representing being polite and friendly and 4 severe aggressiveness), and global assessment of functioning (GAF, 0–100, with 0 representing the extremely psychiatrically ill patient, and 100 the extremely psychiatrically healthy person (adapted from Bodlund [17]). All rating scales contained anchor points.

We tested inter-rater agreement by having a different rater rate all patients who came to pick up medication on one day (n = 26). Inter-rater agreement was satisfactory for single item instruments (range for intra-class correlation: 0.39 to 0.53). We also had the treating physician at the clinic rate a total of 26 patients using the clinical global impression scale for substance use problems and externalizing behaviour, and rate the GAF, whilst being blind to the nurses ratings. Analyses showed moderate associations between physician ratings and nurses ratings (clinical global impression of current substance use problems strongly associated with nurse-rated withdrawal and intoxication: rho = 0.48 and 0.59, p < 0.02; clinical global impression of externalizing behaviour strongly related with nurse-rated externalizing behaviours: rho = 0.41, p = 0.04; physician rated GAF strongly related with nurse rated GAF: rho = 0.62, p < 0.001)).

While the nurses' knowledge of patients varied, they had health check-ups with patients, and regular contact and did some case management with patients, especially in relationship to patients' medical needs.

The nurses and other staff members were kept blind to the results of the SAPAS and other data from the interviews. A total of 5 different nurses rated the patients. As pick-up frequency varied from five days per week to once per two weeks, the number of ratings differed substantially between patients.

### Sample description

A total of 67 patients were approached, and 87% (58) gave consent to participate in the study. Seven declined, and 2 were deemed unable to give consent due to a mixture of language problems and psychiatric problems.

The sample of 58 patients consisted of 42 men and 16 women. The mean age at consent was 39.9 years (SD = 8.97, range = 23 to 62).

Of the 58 patients who gave consent, 48 were in methadone maintenance treatment, and 10 were in Buprenorphine maintenance treatment. Two accepted participation in the study, but were unwilling to be interviewed. Poly-pharmacy was common in the clinic, with 42% receiving benzodiazepines, 22% receiving antidepressants, and 7% receiving antipsychotics. In total, 65% received other medications than opioid substitution. Methadone-doses were 0–40 mg for 3 patients, 41–80 for 13, and over 80 mg for 32.

Patients were asked about their desire to leave opioid substitution maintenance. No desire to quit substitution treatment was reported by 28%, desire to do so some day in an unknown future by 34%, desire to do so within the next 6 months by 26%, and desire to quit substitution as soon as possible by 7%.

A total of 58% had a personality disorder diagnosis according to the SAPAS [16]. However, the SAPAS does not show which personality disorder patients have, and no further assessment of this question was made. The mean proportion of time since the age of 15 that patients reported having worked was 26%. Only two patients were employed at the time of interview.

### Analyses

The reliability of the SAPAS was estimated using Cronbach's alpha, and test-retest reliability of the full scale was estimated using intra-class correlation. For each item, we calculated Cohen's κ as a chance-corrected measure of agreement.

Criterion validity of the SAPAS was estimated using staff ratings of functioning, including withdrawal, intoxication, externalizing behaviour and GAF. We used the mean of all scores over all observations in a 6-month period at the clinic to assess validity. We calculated regular Spearman correlation coefficients, because there were strong deviations from normality in the rating scales (externalizing behaviour and withdrawal had very strong skew.

## Results

### Reliability of the SAPAS

The results of the reliability analyses are shown in Table 1.

The internal consistency of the SAPAS was slightly lower in this sample than in the psychiatric sample (α = 0.62). As in the original study by Moran and colleagues, the impulsivity item reduced reliability slightly. The test-retest reliability of individual items ranged from 0.26 to 0.58. When omitting subjects who were re-interviewed by the same interviewer at both points, the test-retest reliability was slightly decreased for 6 of eight items. For the total SAPAS, the test-retest intraclass correlation was 0.58 (asymptotic z = 2.81, p = 0.005). When omitting subjects who were interviewed by the same interviewer, the intraclass correlation was reduced to 0.52 (asymptotic z = 2.19, p = 0.02).

The mean SAPAS score increased from baseline to follow-up. The mean at baseline was 3.3 (standard deviation = 2.0), and the mean at follow-up was 3.8 (standard deviation = 1.7). The increase was marginally significant (t = -1.99, p = 0.06).

### Criterion validity of the SAPAS

In order to assess the validity of the SAPAS, we used the nurse ratings of functioning. As pick-up frequency varied between individuals, the number of ratings that could be included varied from four to 48 with a mean of 20.1.

The convergent validity correlations are shown in Table 2. The correlations with withdrawal and intoxication were non-significant, but the correlations with externalizing behaviour (rho = 0.38, p < 0.01) and Global Assessment of Functioning (rho = -0.29, p = 0.03) were significant.

**Table 2:** Convergent validity of the SAPAS

	Correlation with SAPAS (Spearman Rho)	P
Withdrawal	0.20	0.13
Intoxication	-0.00	0.99
Externalizing behaviour	0.38	0.00
Global Assessment of Functioning	-0.29	0.03

For further illustration, the means and standard deviation of nurse ratings on the four indicators by SAPAS score are shown in Table 3. The mean score on externalizing behaviour is 0.0 for the single patient scoring 0 on the SAPAS, but it is clear that the mean score increases as the number of SAPAS items endorsed increase. Similarly, the GAF score of patients scoring <3 on the SAPAS are around 60, but if the score is 6–7, the GAF score is around four points lower (55–56).

**Table 3:** Nurse ratings by SAPAS scores

Scores on the SAPAS		Withdrawal		Intoxication		Externalizing behaviour		Global Assessment of Functioning	
	N	Mean	SD	Mean	SD	Mean	SD	Mean	SD

0	1	0.00	0.00	0.00	0.00	0.00	0.00	65.00	0.00
1	13	0.28	0.18	0.71	0.39	0.12	0.12	60.32	3.97
2	10	0.20	0.11	0.80	0.58	0.09	0.10	61.05	3.97
3	8	0.35	0.23	0.78	0.60	0.15	0.17	60.69	6.62
4	9	0.20	0.11	0.50	0.38	0.17	0.15	60.32	2.37
5	6	0.24	0.21	0.65	0.39	0.18	0.18	58.84	5.34
6	3	0.50	0.04	1.05	0.75	0.25	0.29	55.85	7.44
7	6	0.45	0.25	0.64	0.32	0.36	0.23	56.61	5.70

Total	56	0.28	0.19	0.69	0.47	0.16	0.17	59.79	4.80

Notes: SD: Standard deviation.

We conducted a multiple regression analysis to control for age and gender, after rank-order transforming the rating scales. The results were similar: Externalizing behaviour (beta = 0.37, t(52) = 2.94, p = 0.004), and GAF (beta = -0.29, t = 2.19, p = 0.03) remained significantly associated with SAPAS.

As the number of observations differed due to variability in pick-up frequency, we also conducted regression analyses controlling for the number of observations that had been recorded in the clinic. Again, the results were highly similar: again, externalizing behaviour (beta = 0.43, t(53) = 3.49, p = 0.001), and GAF (beta = -0.29, t(53) = -2.52, p = 0.01) remained substantially correlated with SAPAS score.

### Other correlates of the SAPAS

No gender difference was found on the SAPAS (men mean: 3.1, SD = 1.9; women mean: 3.7, SD = 2.3, t(54) = -0.99, p = 0.33), and the correlation between age and SAPAS was non-significant (Pearson r = 0.13, NS). Also, no difference was found between patients who were receiving methadone substitution and patients who were receiving buprenorphine substitution (methadone: 2.28, SD = 2.1; buprenorphine: 3.2, SD = 1.7; t = 0.07, p = 0.94), and no association was found between methadone dose and SAPAS score among those in methadone maintenance treatment (r = 0.11, p = 0.46, n = 46).

Total SAPAS score was negatively correlated with the proportion of time since age 15 that had been spent working without interruption (Spearman rho = -0.31, p = 0.02), but unrelated to degree of desire to quit methadone (Spearman rho = -0.07, p = 0.60).

## Discussion

The findings of this study supported the validity of the SAPAS, and gave indications of modest longer-term stability. Even when different interviewers interviewed the same patients 4 months ago, the stability coefficient was reasonable for such a short measure.

Patients who reported more features of personality disorders, were rated as displaying more externalizing behaviour was observed by the nurses (unsatisfied, complaining, angry), and as having lower scores on the Global Assessment of Functioning. In contrast with our expectations, we found no strong indication of higher ongoing substance use during treatment with personality disorders. However, in this clinic, most patients use drugs and alcohol to some extent, and stable abstinence is rare.

We did not use another interview or self-report inventory to assess the validity of the SAPAS. Obviously, this means that we do not know how many of these patients would have been diagnosed with another instrument. Although such a replication of the original findings of Moran and colleagues could potentially be important, this study adds new light to the validity of this very brief instrument: the SAPAS identifies patients with problems that are highly indicative of personality disorder. It identifies patients who show more aggressive behaviour, and who appear to be functioning less well in terms of their mental health.

The SAPAS was constructed based on primary care patients, and its applicability was originally tested on psychiatric patients [16]. Its items includes item that reflect content related primarily to cluster A personality (being a loner), one item relating to both cluster A and B (trusts people, reversed scored), two items relating to cluster B disorders (impulsive and problems controlling temper), and four items relating primarily to cluster C disorders (difficulty getting and keeping friends; usually dependent; usually a worrier; perfectionist), and thus has a reasonably broad coverage of the various personality disorders in the DSM-IV.

In terms of clinical use, the SAPAS may alert clinicians to the possibility of personality disorder in a patient, but will obviously never serve as a substitute for a full assessment of personality disorders.

### Several strengths and limitations deserve comment

The first strength of the study is validation through independent and blinded assessment by multiple observers over an extended period of time, in that several different nurses rated the same patients every time they came to pick up medications. Patients may fluctuate from day to day, from week to week, depending on events that occur in their life, and nurses may differ in their views of particular patients. However, when observations are averaged over several observers and several occasions over a long period of time, these sources of error is levelled out.

Limitations include a limited sample size, and the use of a cross-sectional sample, rather than a consecutive sample. Also, the validity correlations are likely to be attenuated as a result of the relative unreliability of the SAPAS.

## Conclusion

In conclusion, there is evidence that this very brief measure gives some indication of the presence of personality disorder. It may be useful as an indicator of the presence of personality disorder, either for screening purposes in a clinical setting, or as a tool in a survey.

## Competing interests

The author(s) declare that they have no competing interests.

## Authors' contributions

MH planned the study, instructed the data collection, interviewed most of the patients at baseline, and designed the outcome measurement scales. JR coordinated the nurse ratings, and personally conducted over one third of the ratings. MKP conducted and organized most of the follow-up interviews. MH drafted the manuscript, and conducted statistical analyses. All three authors read and approved of the final manuscript.

## Pre-publication history

The pre-publication history for this paper can be accessed here:



## References

[B1] Grant BF, Stinson FS, Dawson DA, Chou PS, June Ruan W, Pickering R (2004). Co-occurrence of 12-month alcohol and drug use disorders and personality disorders in the United States. Results from the National Epidemiologic Survey of Alcohol and Related Conditions. Archives of General Psychiatry.

[B2] Verheul R (2001). Co-morbidity of personality disorders in individuals with substance use disorders. European Psychiatry.

[B3] Betan E, Heim AK, Zittel Conklin C, Westen D (2005). Countertransference phenomena and personality pathology in clinical practice: an empirical investigation. American Journal of Psychiatry.

[B4] Grella CE, Joshi V, Hser YI (2003). Followup of cocaine-dependent men and women with antisocial personality disorder. Journal of Substance Abuse Treatment.

[B5] Galen LW, Brower KJ, Gillespie BW, Zucker RA (2000). Sociopathy, gender, and treatment outcome among outpatient substance abusers. Drug Alcohol Depend.

[B6] Kranzler HR, Tennen H, Babor TF, Kadden RM, Rounsaville BJ (1997). Validity of the longitudinal, expert, all data procedure for psychiatric diagnosis in patients with psychoactive substance use disorders. Drug Alcohol Depend.

[B7] Kokkevi A, Stefanis N, Anastasopoulou E, Kostogianni C (1998). Personality disorders in drug abusers: prevalence and their association with AXIS I disorders as predictors of treatment retention. Addictive Behaviors.

[B8] Conrod PJ, Stewart SH, Pihl RO, Cote S, Fontaine V, Dongier M (2000). Efficacy of brief coping skills interventions that match different personality profiles of female substance abusers. Psychology of Addictive Behaviors.

[B9] Ball SA, Cobb-Richardson P, Connolly AJ, Bujosa CT, O'Neall T W (2005). Substance abuse and personality disorders in homeless drop-in center clients: symptom severity and psychotherapy retention in a randomized clinical trial. Comprehensive Psychiatry.

[B10] Nielsen P, Røjskjær S, Hesse M (2007). Personality-guided treatment for alcohol dependence: A quasi-randomized experiment. American Journal on Addictions.

[B11] Craig RJ (2000). Prevalence of Personality Disorders among Cocaine and Heroin Addicts. Substance Abuse.

[B12] Hesse M, Nielsen P, Røjskjær S (2007). Stability and Change in Millon Clinical Multiaxial Inventory II Personality Disorder Scores in Treated Alcohol Dependent Subjects: Relationship to Post-treatment Abstinence. International Journal of Mental Health and Addiction.

[B13] Calsyn DA, Wells EA, Fleming C, Saxon AJ (2000). Changes in Millon Clinical Multiaxial Inventory scores among opiate addicts as a function of retention in methadone maintenance treatment and recent drug use. American Journal of Drug and Alcohol Abuse.

[B14] Langbehn DR, Pfohl BM, Reynolds S, Clark LA, Battaglia M, Bellodi L, Cadoret R, Grove W, Pilkonis P, Links P (1999). The Iowa Personality Disorder Screen: development and preliminary validation of a brief screening interview. Journal of Personality Disorder.

[B15] Van Horn E, Manley C, Leddy D, Cicchetti D, Tyrer P (2000). Problems in developing an instrument for the rapid assessment of personality status. European Psychiatry.

[B16] Moran P, Leese M, Lee T, Walters P, Thornicroft G, Mann A (2003). Standardised Assessment of Personality - Abbreviated Scale (SAPAS): preliminary validation of a brief screen for personality disorder. British Journal of Psychiatry.

[B17] Bodlund O, Kullgren G, Ekselius L, Lindstrom E, von Knorring L (1994). Axis V--Global Assessment of Functioning Scale. Evaluation of a self-report version. Acta Psychiatrica Scandinavica.

